# Both Light-Induced SA Accumulation and ETI Mediators Contribute to the Cell Death Regulated by *BAK1* and *BKK1*

**DOI:** 10.3389/fpls.2017.00622

**Published:** 2017-04-25

**Authors:** Yang Gao, Yujun Wu, Junbo Du, Yanyan Zhan, Doudou Sun, Jianxin Zhao, Shasha Zhang, Jia Li, Kai He

**Affiliations:** Ministry of Education Key Laboratory of Cell Activities and Stress Adaptations, School of Life Sciences, Lanzhou UniversityLanzhou, China

**Keywords:** Arabidopsis, *BAK1*, *BKK1*, cell death, light, SA, PTI, ETI

## Abstract

Receptor-like kinases BAK1 and BKK1 modulate multiple cellular processes including brassinosteroid signaling and PRR-mediated PTI in Arabidopsis. Our previous reports also demonstrated that *bak1 bkk1* double mutants exhibit a spontaneous cell death phenotype under normal growth condition. With an unknown mechanism, the cell death in *bak1 bkk1* is significantly suppressed when grown in dark but can be quickly induced by light. Furthermore, little is known about intrinsic components involved in *BAK1* and *BKK1*-regulated cell death pathway. In this study, we analyzed how light functions as an initiator of cell death and identified ETI components to act as mediators of cell death signaling in *bak1 bkk1*. Cell death suppressed in *bak1 bkk1* by growing in dark condition recurred upon exogenously treated SA. SA biosynthesis-related genes *SID2* and *EDS5*, which encode chloroplast-localized proteins, were highly expressed in *bak1-4 bkk1-1*. When crossed to *bak1-3 bkk1-1, sid2* or *eds5* was capable of efficiently suppressing the cell death. It suggested that overly produced SA is crucial for inducing cell death in *bak1 bkk1* grown in light. Notably, *bak1-3* or *bkk1-1* single mutant was shown to be more susceptible but *bak1-3 bkk1-1* double mutant exhibited enhanced resistance to bacterial pathogen, suggesting immune signaling other than PTI is activated in *bak1 bkk1*. Moreover, genetic analyses showed that mutation in *EDS1* or *PAD4*, key ETI mediator, significantly suppressed the cell death in *bak1-3 bkk1-1*. In this study, we revealed that light-triggered SA accumulation plays major role in inducing the cell death in *bak1 bkk1*, mediated by ETI components.

## Introduction

Plant cells utilize plasma membrane (PM)-localized receptor-like kinases (RLKs) to communicate with each other and respond to environmental challenges in coordinating plant growth, development and stress adaptions. RLKs, the single-pass transmembrane proteins with an extracellular domain and a cytoplasmic kinase domain, play essential roles in transducing apoplastic signals into intracellular responses. Leucine-rich repeat (LRR)-RLK family is the largest RLK subfamily containing at least 223 members (Gou et al., 2010), a number of which were found to regulate a variety of physiological processes, such as phytohormone signaling (Li and Chory, 1997; Li et al., 2002; Nam and Li, 2002), innate immunity (Gómez-Gómez and Boller, 2000; Zipfel et al., 2006), meristem maintenance (Clark et al., 1997), pollen development (Zhao et al., 2002; Albrecht et al., 2005; Colcombet et al., 2005), stomatal development (Shpak et al., 2005), pollen tube-egg recognition (Escobar-Restrepo et al., 2007) and cell death control (He et al., 2007, 2008; Kemmerling et al., 2007) etc. Arabidopsis SOMATIC EMBRYOGENESIS RECEPTOR-LIKE KINASE (SERK) family, containing SERK1-SERK5, has become one of the best functionally analyzed LRR-RLK subfamilies (Li, 2010). *SERK* was originally isolated in carrot and identified as a marker gene of somatic embryogenesis (Schmidt et al., 1997). In Arabidopsis, *SERKs* were found to regulate various signaling pathways, in which they often play redundant roles. *SERK1* and *SERK2* regulate porogenesis by maintaining a normal tapetum during pollen maturation (Albrecht et al., 2005; Colcombet et al., 2005); SERK1, SERK3/BRI1-ASSOCIATED KINASE 1 (BAK1), and SERK4/BAK1-LIKE 1 (BKK1) act as co-receptors of the brassinosteroid (BR) receptor BRASSINOSTEROID INSENSITIVE 1 (BRI1) to perceive BRs and initiate cellular signaling cascade (Li et al., 2002; Nam and Li, 2002; Karlova et al., 2006; He et al., 2007; Gou et al., 2012).

BAK1 is also engaged in plant innate immunity. A two-tiered system has been proposed to regulate plant innate immunity (Dodds and Rathjen, 2010). The first layer of immunity is activated after perceiving pathogen-associated molecular patterns (PAMPs) by pattern recognition receptors (PRRs) localized at the surface of a plant cell, known as PAMP-triggered immunity (PTI) (Macho and Zipfel, 2014). The second layer of immunity relies on recognition of pathogen-derived effectors by intracellular resistance (R) proteins in plants, known as effector-triggered immunity (ETI) (Jones and Dangl, 2006). PTI confers plant basal resistance to pathogens and ETI enables faster and stronger defense responses in plants, which is often accompanied with hypersensitive response (HR), a type of programmed cell death (PCD) (Jones and Dangl, 2006; Macho and Zipfel, 2014). Canonical R proteins are nucleotide-binding/leucine-rich repeat (NLR) receptors which fall into two groups, including CNLs, possessing a coiled-coil (CC) domain, and TNLs, containing a Toll-Interleukin-1 receptor (TIR) domain (Meyers et al., 1999; Cannon et al., 2002). EDS1 and PAD4, two lipase-like proteins, are required for TNL-mediated ETI; while NDR1, a PM-associated protein, functions as key mediator in CNL signaling (Aarts et al., 1998; Wiermer et al., 2005; Knepper et al., 2011). BAK1 was found to be involved in PAMP recognition and mediating PTI via associating with several PRR RLKs such as FLAGELLIN-SENSITIVE 2 (FLS2), the receptor for bacterial flagellin, EF-TU RECEPTOR (EFR), the receptor for bacterial elongation factor Tu (EF-Tu), and PEP1 RECEPTOR 1 (PEPR1), the receptor for plant endogenous PROPEP-derived Pep epitopes (Chinchilla et al., 2007; Heese et al., 2007; Schulze et al., 2010). *BAK1*, therefore, positively regulates PTI, the first tier of plant innate immunity.

Intriguingly, our previous studies revealed *BAK1*/*SERK3* and *BKK1*/*SERK4* also mediate a cell death pathway that is independent of the BR signaling pathway (He et al., 2007). A null double mutant, *bak1-4 bkk1-1*, exhibits an extremely strong cell lesion phenotype even on the sterilized medium under normal growth condition. The cell death symptom of *bak1-4 bkk1-1* emerged about 7 days after germination and eventually led to lethality within 2 weeks (He et al., 2007). The cell death phenotype we reported was in line with a simultaneous study of a different group showing that *bak1-4* single mutant exhibited a runaway cell death upon microbial pathogen inoculation (Kemmerling et al., 2007).

Salicylic acid (SA) was identified as an essential signal molecule in mediating plant defense responses (Vlot et al., 2009). Serving as a crucial signal for activating disease resistance including PTI and ETI, SA is rapidly synthesized upon pathogen invasions. Arabidopsis mutants that fail to sufficiently synthesize endogenous SA exhibit impaired innate immunity and enhanced susceptibility to pathogen treatments (Rogers and Ausubel, 1997; Nawrath and Métraux, 1999; Wildermuth et al., 2001; Nawrath et al., 2002). Given that *PR1* is a key marker gene of SA signaling, the extremely high expression of *PR1* suggested an exceedingly activated SA signaling in *bak1-4 bkk1-1* (He et al., 2007). In Arabidopsis, SA is synthesized through two distinct routes, the phenylalanine ammonia-lyase (PAL) pathway and the isochorismate (IC) pathway (Dempsey et al., 2011). Chorismate, the end product of the shikimate pathway, is used as the SA precursor in both pathways (Dempsey et al., 2011). In Arabidopsis, four PAL proteins (PAL1-4) are essential for catalyzing the reaction from phenylalanine to trans-cinnamic acid, a critical step for SA production (Mauch-Mani and Slusarenko, 1996; Chen et al., 2009; Huang et al., 2010). The PAL pathway occurs in cytoplasm and was proposed to account for only 5% of SA production (Métraux, 2002; Chen et al., 2009). The IC pathway, by contrast, takes place in chloroplast and contributes 95% of total cellular SA production (Wildermuth et al., 2001; Métraux, 2002; Garcion et al., 2008; Chen et al., 2009). Two redundant genes *ISOCHORISMATE SYNTHASE 1* (*ICS1*) and *ICS2* were identified to encode essential enzymes localized in chloroplasts to catalyze the conversion of chorismate to isochorismate, a key step of SA biosynthesis in the IC pathway (Garcion et al., 2008). Since *ICS2* only plays a marginal role in this reaction, *ICS1*, also known as *SALICYLIC ACID INDUCTION DEFICIENT 2* (*SID2*), functions as the determinate catalyzator of SA synthesis in IC pathway (Wildermuth et al., 2001; Garcion et al., 2008). Distinct from WT, *ics1*/*sid2* single mutant plants showed no obvious SA accumulation upon a variety of stress treatments such as UV light, ozone, or pathogen incubations (Nawrath and Métraux, 1999; Garcion et al., 2008). The majority of SA, therefore, appears to be produced in chloroplasts and subsequently transported to cytosol and other cellular compartments to fulfill its physiological roles. It has been previously reported that ENHANCED DISEASE SUSCEPTIBILITY 5 (EDS5), a member of Multidrug and Toxic Compound Extrusion (MATE) transporter family, also impacts SA accumulation (Nawrath et al., 2002). EDS5 was suggested to be localized on plastid membrane and presumably function as an SA transporter in translocating SA from chloroplasts to cytosol (Serrano et al., 2013). Like *ics1*/*sid2, eds5* mutants also failed to efficiently accumulate SA when exposed to pathogens (Nawrath and Métraux, 1999).

Little progress has been made since *BAK1* and *BKK1* were discovered to be essential in mediating a cell death pathway. One earlier study indicated that another LRR-RLK, BAK1-INTERACTING RECEPTOR-LIKE KINASE 1 (BIR1), can interact with BAK1, and that *bir1* mutants displayed a cell death phenotype similar to that of the *bak1 bkk1* double mutants (Gao et al., 2009). A PM-localized copine-like protein, BONZAI1 (BON1), was identified as BAK1- and BIR1-interacting protein (Wang et al., 2011). The *bon1* mutant plants also exhibited a cell-death phenotype (Yang et al., 2006). However, the detailed mechanisms of *BIR1* and *BON1* in regulating cell death pathways and their connections to the *BAK1* and *BKK1*-regulated cell death pathway remain elusive. Key open questions still await answers. For instance, what are the primary factors responsible for initiating cell death in *bak1 bkk1* double mutant at early stage? Which biological processes are related to the cell death of *bak1 bkk1*? Our previous report suggested that light likely serves as a key factor in triggering cell death in *bak1-4 bkk1-1* (He et al., 2008). Distinct from the *bak1-4 bkk1-1* plants starting to exhibit lesion symptom at 7 days when grown under a long-day condition, *bak1-4 bkk1-1* grown in darkness did not display a cell death phenotype at 8 days (He et al., 2008). The mechanism regarding how light contributes to triggering cell death in *bak1 bkk1* has not been depicted. Despite the well establishment of BAK1 as an essential co-receptor of key PRRs in positively regulating PTI, the cell death occurring in *bak1 bkk1* seems to be unrelated to PTI. Overexpression or knocking-out of known BAK1-associated PRRs does not cause cell death in plants. Instead, the cell death phenotype of *bak1-4 bkk1-1* resembles HR, a typical defense response in ETI. It implies *BAK1* and *BKK1* play roles beyond PTI. However, whether ETI regulatory components are involved in the cell death pathway mediated by *BAK1* and *BKK1* remains unknown.

In this study, we carefully analyzed the light-triggered cell death in *bak1 bkk1* mutants. Our results indicated that SA accumulation in *bak1 bkk1* mutants may function as a primary initiator to trigger cell death. Genes encoding chloroplast-localized proteins SID2 and EDS5 that are essential for SA biosynthesis and accumulation were highly up-regulated in *bak1 bkk1* and played critical roles in cell death formation. Further analyses revealed that, despite maintaining their insensitivity to bacterial PAMP, *bak1 bkk1* mutants exhibited overly activated immunity, suggesting *BAK1* and *BKK1* mediate a different immunity signaling besides PTI. At last, our results showed that the loss-of-function mutation of *PAD4* or *EDS1*, key component mediating ETI, was capable of significantly suppressing the cell death of a weak double mutant, *bak1-3 bkk1-1*. These results provide genetic evidence suggesting the involvement of ETI in *BAK1* and *BKK1*-mediated cell death pathway.

## Materials and methods

### Plant materials and growth conditions

All of the Arabidopsis lines used in this study are in the Col-0 ecotype except *pif1-1* (Col-3). T-DNA insertion lines *bak1-4* (SALK_116202), *bak1-3* (SALK_034523), *bkk1-1* (SALK_057955), *sid2-3* (SALK_088254), *eds5-2* (SALK_091541C), *pad4*-2 (SALK_089936), *eds1-3* (SALK_057149), *pif1-1* (CS66041), *pif3-1* (SALK_030753), *pif4-3* (SALK_140393C), *pif5-3* (SALK_087012C) were obtained from the Arabidopsis Biological Resource Center (ABRC). Multiple mutants were generated by genetic crossings. Homozygous plants were isolated via PCR with genotyping primers designed by http://signal.salk.edu/tdnaprimers.2.html. The wildtype and mutant plants were grown in greenhouse or illumination incubator at 22°C in dark condition or under long day cycle (16 h light/8 h dark). For SA treatment assay, seedlings were grown on 0.5 × Murashige and Skoog medium with or without SA in dark condition and were analyzed at indicated days.

### Trypan blue staining and DAB staining

Tissue stainings with trypan blue and DAB were carried out as previously described (He et al., 2007).

### Determination of subcellular distribution

The full length CDS of *SID2* and *EDS5* were amplified by RT-PCR and cloned into pBIB-BASTA-35S-GWR-GFP and pBIB-BASTA-35S-GWR-YFP, respectively. The two constructs were transformed into Agrobacterium tumefaciens strain GV3101. The transformed GV3101 were cultured in LB medium (supplemented 20 μM acetosyringone) about 12–16 h. Centrifuged and re-suspended the cultured cells with injection buffer (liquid MS medium containing 150 μM acetosyringone, 10 mM MgCl_2_, 10 mM MES, pH 5.7) and adjusted to OD_600_ = 0.5. After quiescence for 2 h, the cultures were injected into the *N. benthamiana* mesophyll tissue. *N. benthamiana* protoplasts were isolated after 3 days of injection. YFP or GFP fluorescence signal was observed by Olympus FluoView FV1000 confocal microscope.

### Gene expression analyses

For quantitative RT-PCR (qRT-PCR) assays, total RNA was extracted from the whole seedlings harvested under different time points. Two micrograms of total RNA was reversely transcribed in a 20 μl volume with M-MLV reverse transcriptase (Invitrogen). The fragments of target genes were amplified using SYBR Premix Ex Taq (TaKaRa). The thermal cycling program was 95°C for 30 s, followed by 40 cycles of 95°C for 5 s, 60°C for 30 s. *ACTIN2* (*At3G18780*) was used for normalization the relative expression level of each transcript, and the comparative ΔΔC_T_ method was employed to calculate the relative quantities of each amplified product. For identification of mutant alleles, total RNA was isolated from the leaves of 11-day-old plants grown in soil. Two micrograms of total RNA was reversely transcribed in a 20 μl volume using M-MLV reverse transcriptase (Invitrogen). The transcripts of specific genes were amplified with gene-specific primers. *ACTIN2* was used as a reference. The primers used for qRT-PCR and semi-quantitative RT-PCR are listed in Table S1.

### Measurements of SA content

SA content measurements were carried out as previously described (Du et al., 2016).

### Bacterial infection assays

*Pst* DC3000-LUX strain expressing *luxCDABE* operon was cultured in KB medium for 12 h. Centrifuged and re-suspended the cultured cells with 10 mM MgCl_2_ and adjusted to OD_600_ = 0.2. The *Pst* DC3000-LUX suspension was sprayed onto the 3-week-old Arabidopsis plant lines indicated. All the sprayed plants were kept under a transparent lid to keep high humidity conditions. The plants were checked by a CCD camera to obtain the images of luciferase fluorescence at 30 h after inoculation. The experiments were repeated three times.

### flg22 treatments

Diluted flg22 with sterilized 0.5 × Murashige and Skoog solution (pH5.7) to the concentration of 0.2 μM. Nine-day-old Col-0, *bak1-4 bkk1-1, bak1-4* and *bkk1-1* grown on 0.5 × Murashige and Skoog medium were immersed with flg22 solution for 5, 15, and 30 min. Total protein was extracted from the seedlings harvested under different time points. The total protein extracts were separated by a 10% SDS-PAGE gel for western blot assay. P-p44/42 (Cell Signaling Technology) was used as the antibody. Equal loadings were tested by staining the SDS-PAGE gel with Coomassie brilliant blue G250. The experiments were repeated three times.

## Results

### Initiation of the cell death in *bak1-4 bkk1-1* was light-dependent

**Figure 1:**
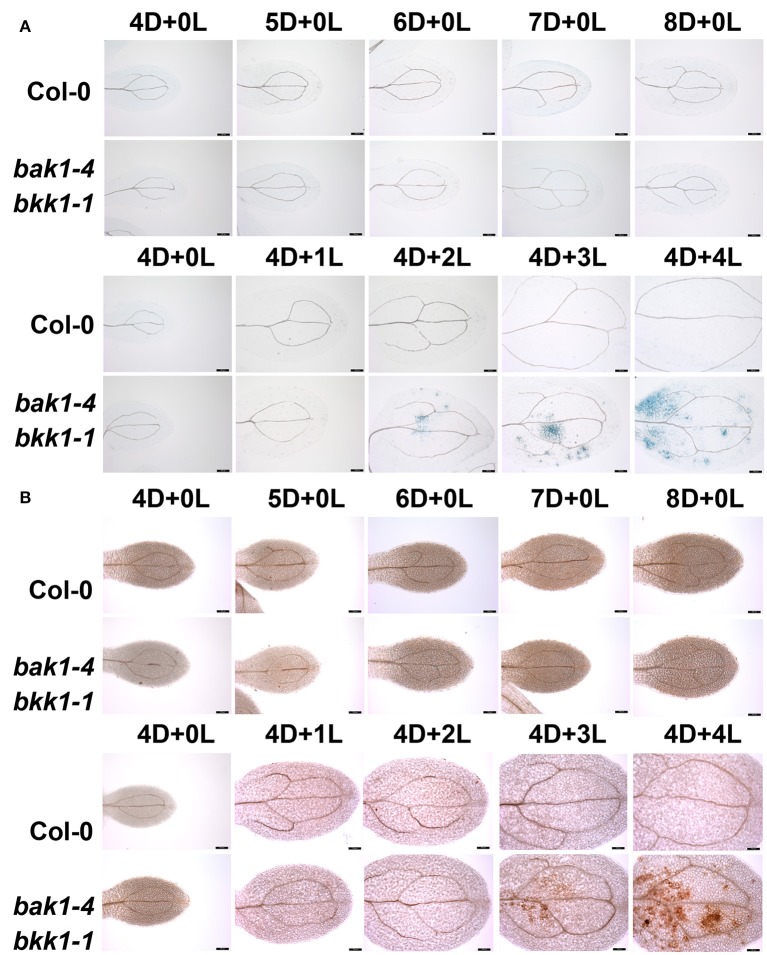
**Light initiates cell death in ***bak1-4 bkk1-1*****. Seedlings are grown in dark (D) for 4 days then relocated to long-day condition (L) for the indicated days (4D+1L, 4D+2L, 4D+3L, 4D+4L). Seedlings grown in dark (D) for the indicated days (4D+0L, 5D+0L, 6D+0L, 7D+0L, 8D+0L) are presented as control. The trypan blue staining assays indicate cell death symptom is induced in *bak1-4 bkk1-1* 2 days after the transfer from dark to light condition **(A)**. The DAB staining assays indicate ROS accumulation is detected in *bak1-4 bkk1-1* 3 days after the transfer from dark to light condition **(B)**. The scale bars represent 100 μm.

In order to elucidate the mechanism of light-induced cell death in *bak1 bkk1*, we analyzed *bak1-4 bkk1-1* mutant plants under a variety of light conditions. Consistent with our earlier results, dark-grown *bak1-4 bkk1-1* showed no clear cell death symptom 8 days after germination, similar to WT Col-0 plants (Figure 1A). Next, Col-0 and *bak1-4 bkk1-1* plants were first grown in dark for 4 days then transferred to a long-day condition (16 h light/8 h dark). *bak1-4 bkk1-1* started to exhibit cell death 2 days after transferring to light (Figure 1A). By contrast, no cell death was detected in dark-grown Col-0 subsequently treated by light (Figure 1A). These results indicated that dark condition prevented *bak1-4 bkk1-1* from forming cell death, which was quickly triggered by light. Cell death is often accompanied with ROS burst, which could be either the cause or consequence of cell death (Chaouch and Noctor, 2010). DAB staining was used to visualize the accumulation of H_2_O_2_. Col-0 did not accumulate H_2_O_2_ under either light or dark condition (Figure 1B). ROS burst was only detected in dark-grown *bak1-4 bkk1-1* after exposure to light (Figure 1B), displaying patterns similar to cell death formation (Figure 1A). Under light condition, the initiation of cell death was found to be 1 day prior to overaccumulation of ROS in *bak1-4 bkk1-1*. These results suggested that, instead of causing cell death, ROS burst may be the physiological consequence of cell death in *bak1-4 bkk1-1*.

**Figure 2:**
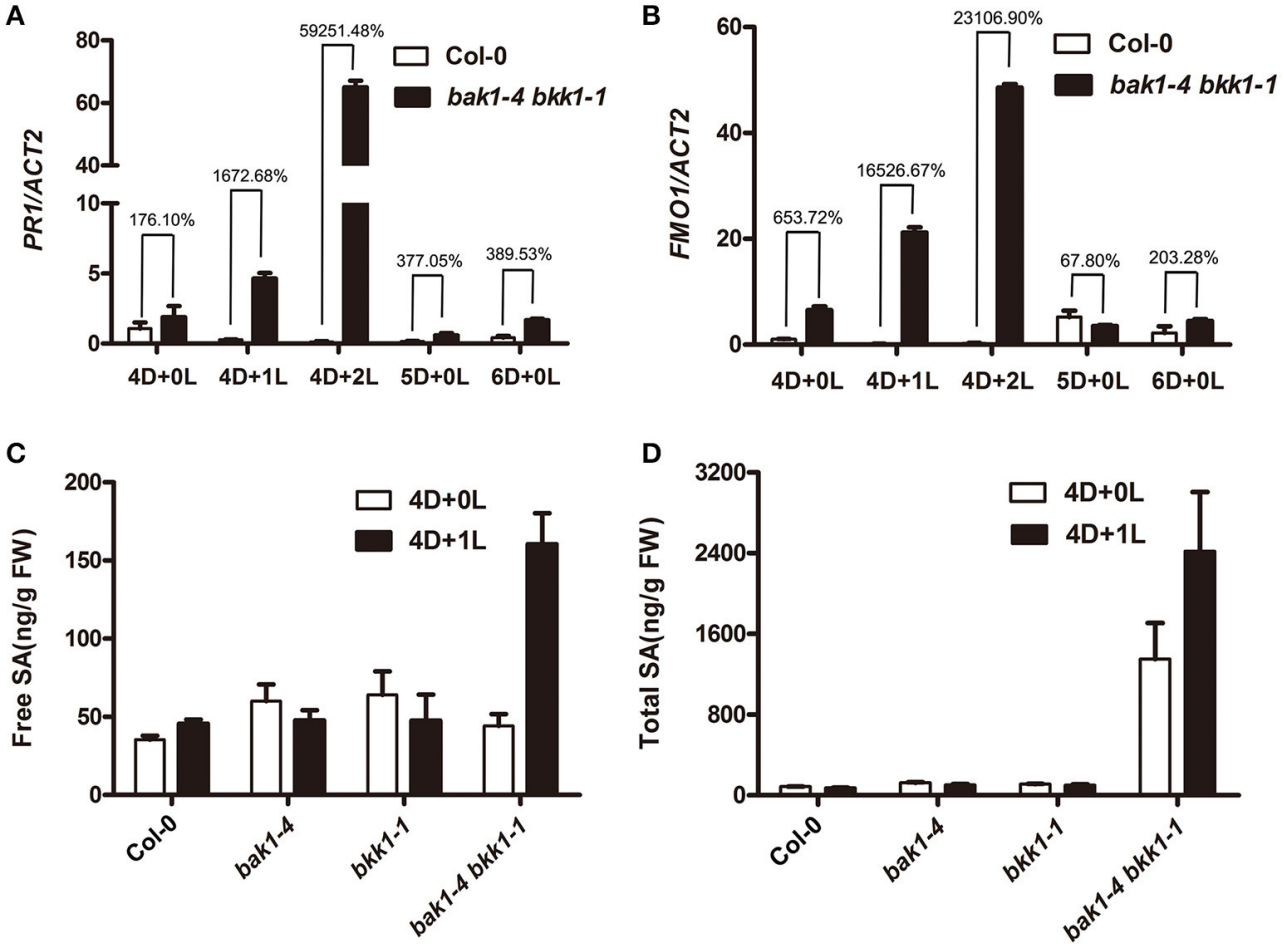
**The transcripts of ***PR1*** and ***FMO1*** and SA content in ***bak1-4 bkk1-1*** are significantly elevated upon light treatment**. Quantitative RT-PCR assays demonstrate the expression of SA marker gene *PR1* **(A)** and cell death marker gene *FMO1* **(B)** is extensively up-regulated in *bak1-4 bkk1-1* by light. Free SA **(C)** and total SA **(D)** contents increase in *bak1-4 bkk1-1* 1 day after the transfer from dark to long-day condition. Seedlings grown in dark (D) for 4 days then relocated to long-day condition (L) for the indicated days (4D+1L, 4D+2L) and seedlings grown in dark (D) for the indicated days (4D+0L, 5D+0L, 6D+0L) are analyzed.

Next, we examined whether the defense response of *bak1-4 bkk1-1* was also related to light. Utilizing qRT-PCR, a SA-responsive gene, *PR1*, and a cell death marker gene, *FOM1* (Bartsch et al., 2006; Li et al., 2013), were checked. Col-0 and *bak1-4 bkk1-1* grown in dark for 4 days then transferred to long-day condition for 1 or 2 days were analyzed. After being transferred to a long-day condition for 1 or 2 days, *PR1* in dark-grown *bak1-4 bkk1-1* increased about 17 or 600-fold, respectively, compared to the one in Col-0 (Figure 2A). In Col-0, *PR1* gene was kept at constantly low expression levels under both dark and light conditions (Figure 2A). *FMO1* expression was also significantly induced when dark-grown *bak1-4 bkk1-1* was exposed to light for 1 or 2 days (Figure 2B), consistent with trypan blue straining results (Figure 1A). As control *PR1* and *FOM1* genes were also analyzed in the seedlings grown in continuous dark. In contrast to the significantly increased expression of *PR1* and *FMO1* in dark-grown *bak1-4 bkk1-1* after light treatment, when grown in dark for 5 or 6 days, the expression of *PR1* and *FMO1* showed slight increases or almost no changes in *bak1-4 bkk1-1* compared to the ones in Col-0 (Figures 2A,B). Since *PR1*, a marker gene of SA signaling pathway, was highly expressed in *bak1-4 bkk1-1* upon light exposure, it was conjectured SA may contribute to the light-induced cell death mediated by *BAK1* and *BKK1*. The SA contents were measured in Col-0, *bak1-4, bkk1-1*, and *bak1-4 bkk1-1* seedlings grown in dark for 4 days and the seedling grown in dark for 4 days followed by exposure to long-day condition for 1 day. The free SA in Col-0 and single mutant plants grown in dark for 4 days was unchanged after transferring to a long-day condition for 1 day. In *bak1-4 bkk1-1* grown under dark for 4 days, free SA increased about 3-fold after growing the plants in long-day condition for 1 day, indicating a quick SA accumulation in *bak1-4 bkk1-1* upon light treatment (Figure 2C). Similarly, total SA in *bak1-4 bkk1-1* plants was also significantly increased when moved from dark to light condition (Figure 2D). Notably, high concentration of total SA was already detected in *bak1-4 bkk1-1* plants grown in dark before light treatment (Figure 2D). This result suggested that *BAK1* and *BKK1* may also regulate a pathway inactivating SA.

The majority of SA is synthesized in chloroplasts where SID2/ICS1 serves as a determinate enzyme (Wildermuth et al., 2001; Métraux, 2002; Garcion et al., 2008; Chen et al., 2009). A minor portion of SA is produced in cytoplasm, which is catalyzed by PALs (Métraux, 2002; Chen et al., 2009). To explicate why total SA was accumulated in *bak1-4 bkk1-1* even under a dark condition, we analyzed the expression levels of *PAL1*-*4* genes. In dark, the expression of all four *PAL* genes increased in *bak1-4 bkk1-1* compared to those in Col-0 (Figure S1). The elevated expression of *PAL* genes in *bak1-4 bkk1-1* may contribute to the accumulation of total SA in a dark condition, when free SA was still kept at low concentrations.

Interestingly, cell death eventually started to be detectable in *bak1-4 bkk1-1* at 12 days when grown in dark (Figure S2). These results indicated that the cell death phenotype was extensively delayed but not completely abolished in *bak1-4 bkk1-1* when grown in dark.

### Exogenously applied SA triggered cell death in *bak1-4 bkk1-1* in dark

**Figure 3:**
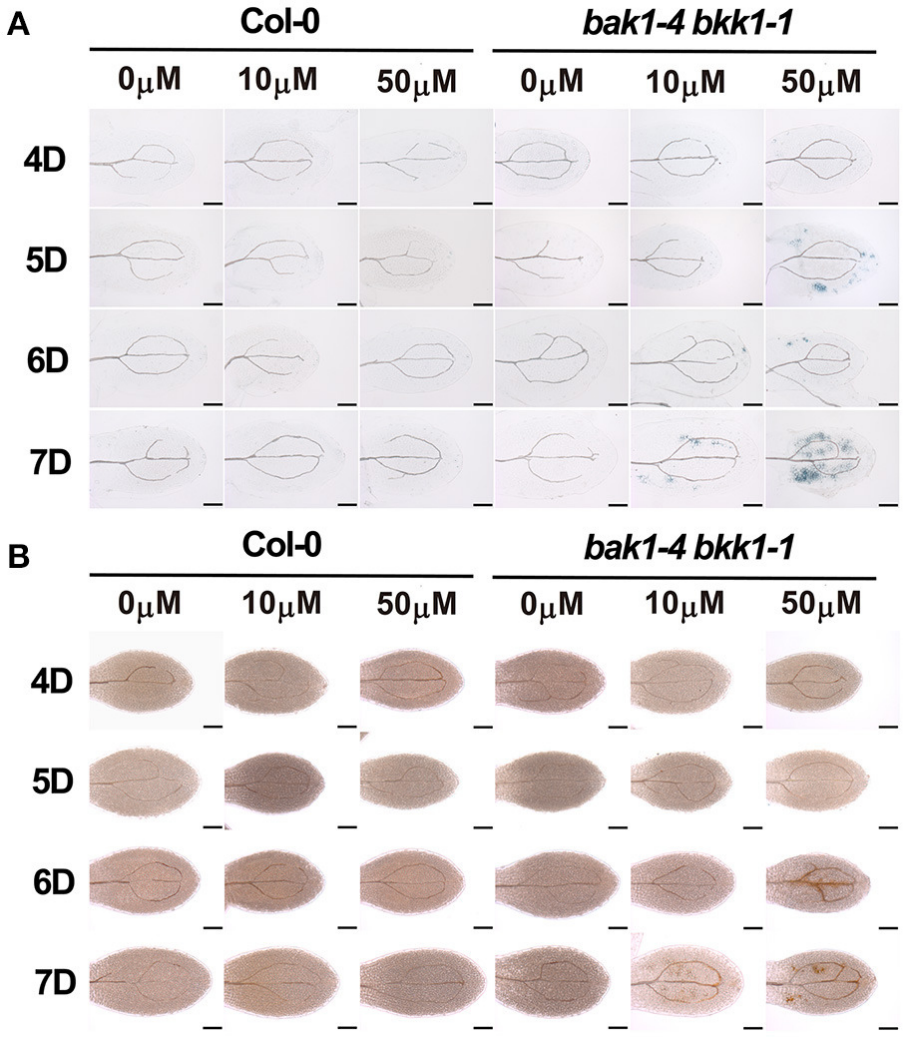
**Exogenously applied SA is sufficient to trigger cell death in ***bak1-4 bkk1-1*** in dark**. The trypan blue staining assays indicate cell death formation in dark-grown *bak1-4 bkk1-1* on the 1/2 MS medium supplemented with 10 or 50 μM SA **(A)**. The DAB staining assays indicate ROS accumulation in dark-grown *bak1-4 bkk1-1* on the 1/2 MS medium supplemented with 10 or 50 μM SA **(B)**. Seedlings grown in dark (D) for the indicated days are presented (4D–7D). The scale bars represent 100 μm.

Given that the initiation of light-dependent cell death in *bak1-4 bkk1-1* was likely SA-related, we further analyzed the detailed role of SA in cell death induction. Trypan blue staining was used to detect cell death in Col-0 and *bak1-4 bkk1-1* grown on 1/2 MS medium supplemented with 0, 10, or 50 μM SA in dark condition. Col-0 showed no cell death when treated with 10 or 50 μM SA (Figure 3A). When grown on the medium supplemented with 50 μM SA in a dark condition, *bak1-4 bkk1-1* started to show cell death symptom 5 days after germination. When treated with 10 μM SA in dark, *bak1-4 bkk1-1* also exhibited obvious but less severe cell death symptom compared to those treated with 50 μM SA (Figure 3A). These results clearly demonstrated that cell death suppressed in *bak1-4 bkk1-1* by growing the seedlings in dark condition reappeared by exogenously treated SA. Therefore, SA seemed to reproduce the effect of light with regard to cell death induction in *bak1-4 bkk1-1*. ROS accumulation was also examined in Col-0 and *bak1-4 bkk1-1* plants upon SA treatment. This result suggested that H_2_O_2_was also accumulated in *bak1-4 bkk1-1* but not in Col-0 when SA was applied to the medium (Figure 3B). In dark condition, the ROS burst in *bak1-4 bkk1-1* appeared 1 day after the emergence of cell death symptom when treated with SA (Figure 3), similar to the patterns of light-induced cell death and ROS burst in *bak1-4 bkk1-1* (Figure 1).

### The expression of *SID2* and *EDS5* was highly elevated in *bak1 bkk1*

**Figure 4:**
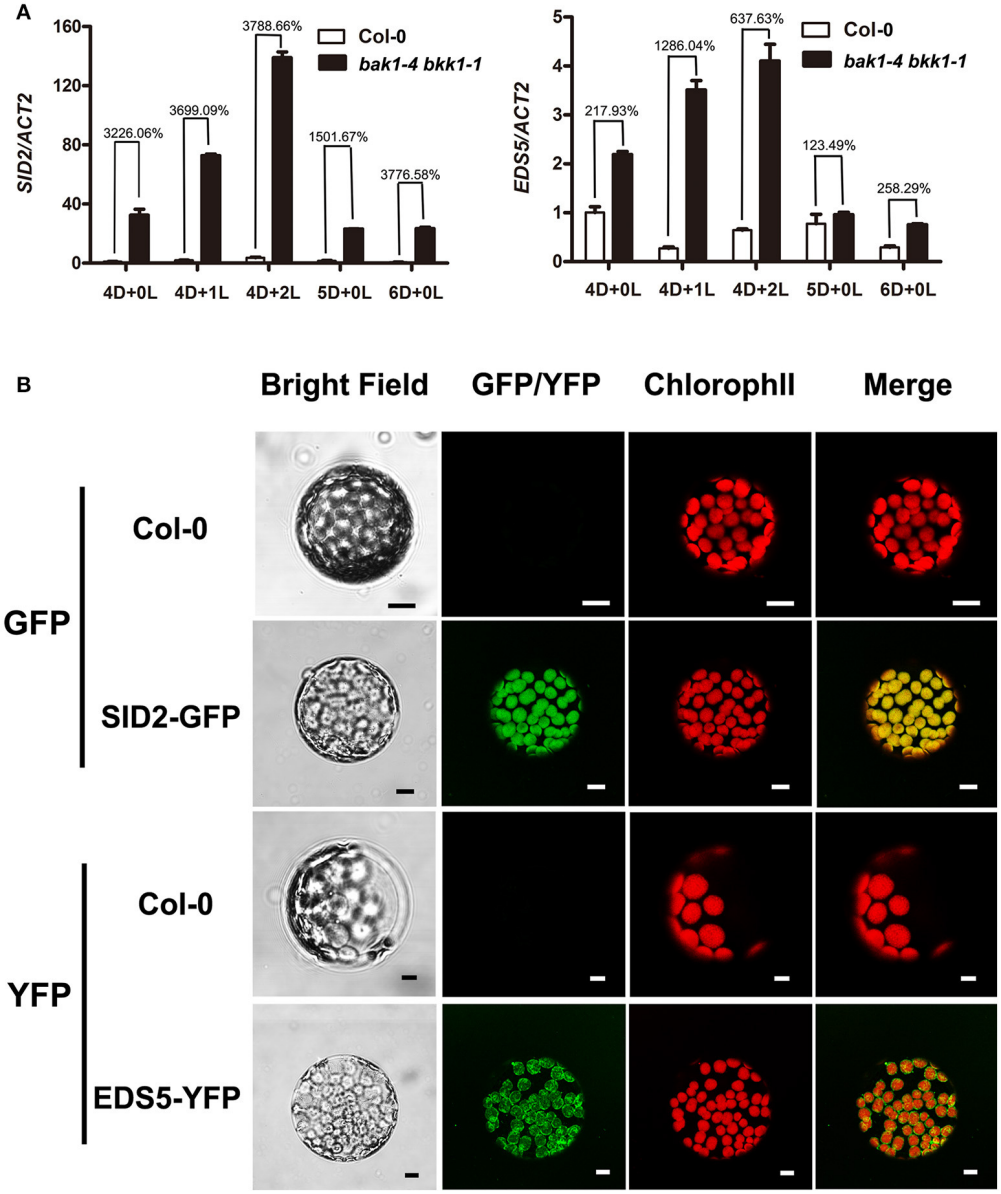
*****BAK1*** and ***BKK1*** robustly suppress the expression of SA biosynthesis-related genes ***SID2*** and ***EDS5*****. Seedlings are grown in dark (D) for 4 days then relocated to long-day condition (L) for the indicated days (4D+0L, 4D+1L, 4D+2L). Seedlings grown in dark for 5 or 6 days (5D+0L or 6D+0L) are analyzed as control. Quantitative RT-PCR assays indicate both *SID2* and *EDS5* are significantly up-regulated in *bak1-4 bkk1-1* compared to those in Col-0 **(A)**. SID2-GFP and EDS5-YFP are detected to locate to the entire chloroplast and chloroplast surface, respectively, in *N. benthamiana* protoplasts **(B)**. The scale bars represent 10 μm.

SA-induced cell death in *bak1-4 bkk1-1* was light-independent, supporting light plays an indirect role in the induction of cell death in *bak1-4 bkk1-1*. SA accumulation may contribute to this process. We first analyzed whether light signaling pathways were involved in *BAK1* and *BKK1*-mediated cell death. A weak double mutant *bak1-3 bkk1-1* was crossed to several *pif* mutants to generate triple and quadruple mutant lines. *PIF* genes encode transcription factors mediating red light and blue light signaling pathways. *PIF1* usually functions redundantly with *PIF3*, and *PIF4* often plays an overlapping role with *PIF5*. Our genetic results demonstrated that none of *bak1-3 bkk1-1 pif1-1, bak1-3 bkk1-1 pif3-1*, or *bak1-3 bkk1-1 pif1-1 pif3-1* mutant plants displayed a phenotypic difference in cell death symptom compared to their background *bak1-3 bkk1-1* (Figure S3). Similarly, *bak1-3 bkk1-1 pif4-3, bak1-3 bkk1-1 pif5-3*, or *bak1-3 bkk1-1 pif4-3 pif5-3* mutant plants did not show alterations to *bak1-3 bkk1-1* with regard to cell death phenotype (Figure S4). These results suggested that the *PIF*-mediated light signalings may not contribute to the cell death initiation in *bak1 bkk1* under light condition. Thus, it was hypothesized that SA synthesis, mainly occurring in chloroplast and relying on light-dependent photomorphogenesis, plays a key role in the light-induced cell death in *bak1 bkk1*. Through data mining of our earlier microarray results that compare global gene expression patterns between Col-0 and *bak1-4 bkk1-1* seedlings, we noticed that two genes, *SID2* and *EDS5*, encoding critical components involved in SA biosynthesis and cytoplasmic accumulation, were dramatically up-regulated in *bak1-4 bkk1-1*. The microarray data indicated that the transcripts of *SID2* and *EDS5* in *bak1-4 bkk1-1* increased 19.86 and 5.78-fold, respectively, compared to the ones in Col-0. Interestingly enough, the protein products of these two genes were both suggested to locate to chloroplast. *SID2* encodes an isochorismate synthase catalyzing chorismate into isochorismate, a SA precursor, in chloroplast. *EDS5* encodes a MATE transporter, locating to the chloroplast envelope, and is suggested to function as an SA transporter mediating the translocation of chloroplast-synthesized SA to cytosol. Using a quantitative RT-PCR assay, we analyzed the expression patterns of *SID2* and *EDS5* in Col-0 and *bak1-4 bkk1-1* plants grown in dark condition for 4 days and the plants grown in dark for 4 days then exposed in long-day condition for 1 or 2 days. *SID2* showed significantly high expression in *bak1-4 bkk1-1* (Figure 4A), indicating a robustly negative regulation on *SID2* expression by *BAK1* and *BKK1*. Treated with or without light condition, the transcripts of *SID2* increased about 30–40-fold in *bak1-4 bkk1-1* compared to those in Col-0 (Figure 4A), suggesting the inhibition on *SID2* expression by *BAK1* and *BKK1* was likely light-independent. In dark, the expression of *EDS5* increased about 2-fold in *bak1-4 bkk1-1* compared to the one in Col-0 (Figure 4A). Upon Light treatment, *EDS5* expression decreased in Col-0 but increased in *bak1-4 bkk1-1* (Figure 4A), suggesting *EDS5* is also suppressed by *BAK1* and *BKK1*, especially under light condition. When grown in dark for 5 or 6 days, *SID2* and *EDS5* in *bak1-4 bkk1-1* showed much lower levels of increases (Figure 4A). We next analyzed the subcellular locations of SID2 and EDS5 proteins. The cDNAs of *SID2* and *EDS5* were fused with *GFP* and *YFP*, respectively, and transiently expressed in *N. benthamiana*, driven by *35S* promoter. SID2-GFP was detected to be localized in chloroplast (Figure 4B). EDS5-YFP mainly located to the surface of chloroplast (Figure 4B), consistent with the previous report that EDS5 may function as an SA transporter at the chloroplast envelope. Taken together, our results indicated *SID2* and *EDS5*, encoding chloroplast-localized proteins essential for SA accumulation, were suppressed by *BAK1* and *BKK1* at transcriptional levels.

### Mutation of *SID2* or *EDS5* partially suppressed the cell death in *bak1-3 bkk1-1*

**Figure 5:**
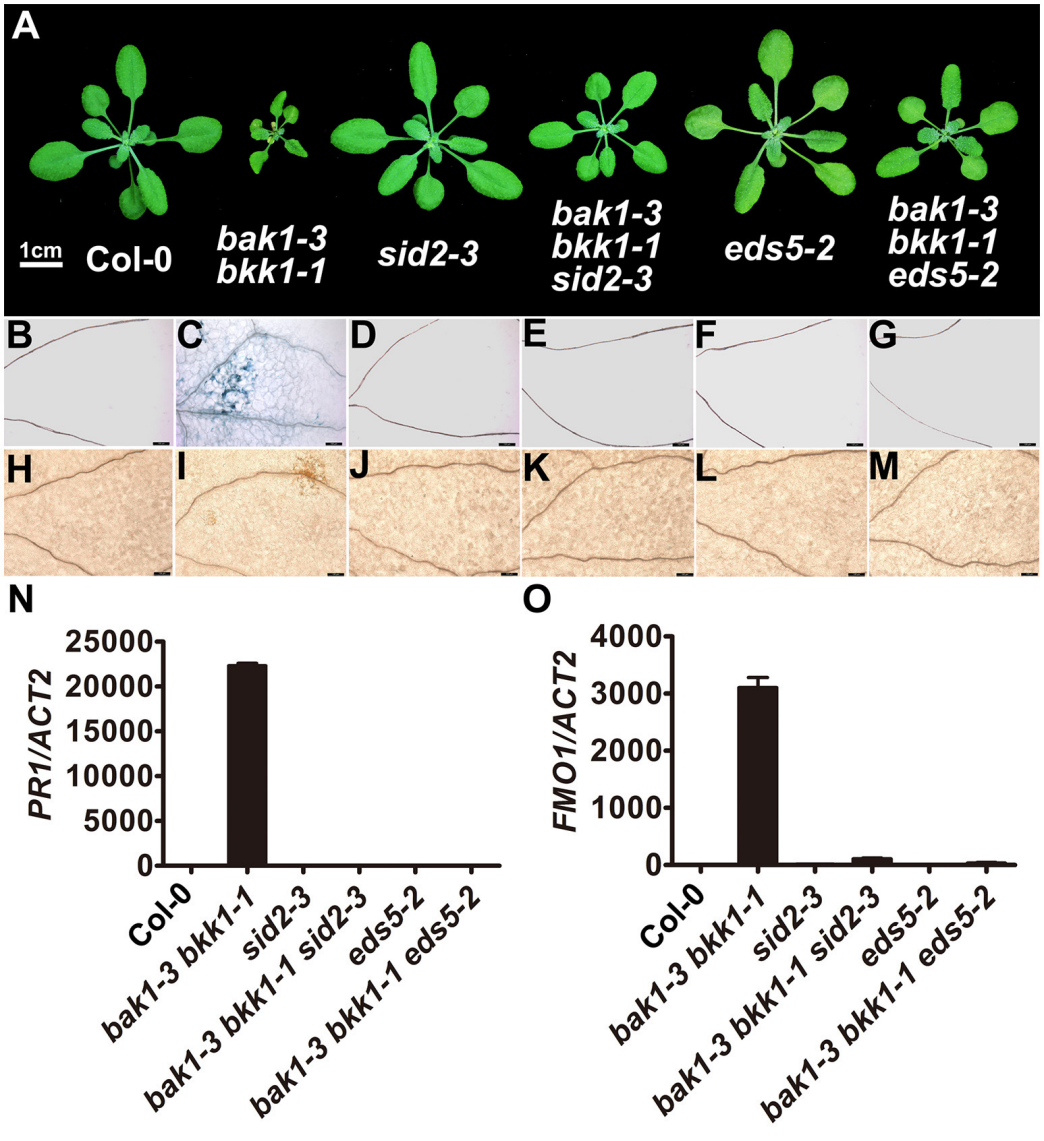
**The cell death in ***bak1-3 bkk1-1*** is significantly suppressed by the mutation in ***SID2*** or ***EDS5*****. The representative phenotypes of 3-week-old Col-0, *bak1-3 bkk1-1, sid2-3, bak1-3 bkk1-1 sid2-3, eds5-2*, and *bak1-3 bkk1-1 eds5-2* grown in soil are presented **(A)**. Trypan blue staining **(B–G)** and DAB staining **(H–M)** assays indicate the cell death and ROS accumulation in *bak1-3 bkk1-1* are largely prevented in *bak1-3 bkk1-1 sid2-3* and *bak1-3 bkk1-1 eds5-2*. Quantitative RT-PCR assays indicate the highly expressed *PR1* and *FMO1* in *bak1-3 bkk1-1* are restored to WT-like levels in *bak1-3 bkk1-1 sid2-3* and *bak1-3 bkk1-1 eds5-2* **(N,O)**. Scale bars represent 1 cm **(A)** and 100 μm **(B–M)**, respectively. 11-day-old seedlings are analyzed in **(B–O)**.

In order to further dissect the function of *SID2* and *EDS5* in initiating cell death in *bak1 bkk1*, genetic approaches were employed. *bak1-3 bkk1-1* was crossed to *sid2-3* and *eds5-2* T-DNA insertion lines, respectively, to generate triple-mutants. RT-PCR results confirmed that *SID2* and *EDS5* were completely knocked out in *bak1-3 bkk1-1 sid2-3* and *bak1-3 bkk1-1 eds5-2*, respectively (Figure S5). Introduction of *sid2-3* into *bak1-3 bkk1-1* significantly suppressed the cell death symptom of *bak1-3 bkk1-1* when grown in soil for 3-week-old (Figure 5A). Trypan blue and DAB staining results confirmed *SID2* mutation greatly inhibited the cell death phenotype and ROS accumulation in 11-day-old *bak1-3 bkk1-1* (Figures 5B–E,H–K). In addition, the highly expressed *PR1* and *FMO1* in *bak1-3 bkk1-1* restored to WT-like levels in *bak1-3 bkk1-1 sid2-3* triple mutant, indicating *SID2* is essential for cell death induction in *bak1-3 bkk1-1* (Figures 5N,O). Similarly, the absence of *EDS5* also appeared to partially rescue the cell death phenotype of 3-week-old *bak1-3 bkk1-1* (Figure 5A). Trypan blue and DAB staining results verified that the cell death and ROS burst in 11-day-old *bak1-3 bkk1-1 eds5*-*2* was largely prevented (Figures 5B–M). The qRT-PCR results showed that the expression of *PR1* and *FMO1* was greatly suppressed in *bak1-3 bkk1-1 eds5-2* compared to the ones in *bak1-3 bkk1-1* (Figures 5N,O). Collectively, our genetic results indicated SA biosynthesis-related genes *SID2* and *EDS5* act as crucial components in promoting cell death in *bak1-3 bkk1-1*.

### *bak1 bkk1* showed enhanced bacterial resistance and impaired PTI

**Figure 6:**
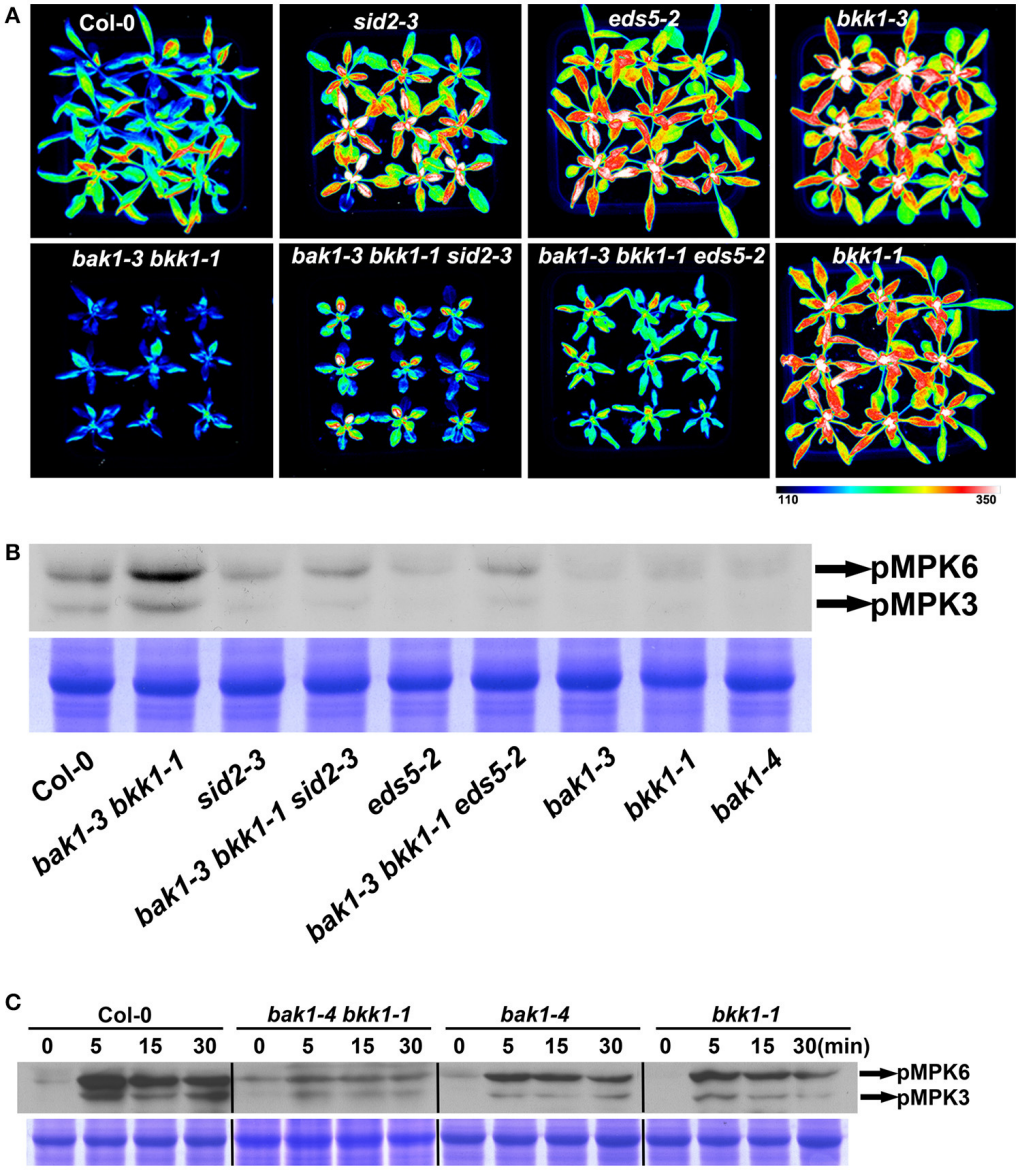
*****bak1-3 bkk1-1*** shows enhanced resistance to bacterial pathogen**. The image of 3-week-old Col-0, *bak1-3 bkk1-1, sid2-3, bak1-3 bkk1-1 sid2-3, eds5-2, bak1-3 bkk1-1 eds5-2, bak1-3*, and *bkk1-1* grown in soil is presented at 30 h after sprayed with *Pst* DC3000-LUX **(A)**. The western blot assay shows the phosphorylation levels of MPK3/MPK6 in 2-week-old Col-0, *bak1-3 bkk1-1, sid2-3, bak1-3 bkk1-1 sid2-3, eds5-2, bak1-3 bkk1-1 eds5-2, bak1-3, bak1-4*, and *bkk1-1* **(B)**. The western blot assay shows the phosphorylation levels of MPK3/MPK6 in 9-day-old Col-0, *bak1-4 bkk1-1, bak1-4*, and *bkk1-1* treated with flg22 **(C)**. Seedlings are grown in soil **(B)** and 0.5× Murashige and Skoog medium **(C)**, respectively.

Since it is established that BAK1 positively regulates PTI via interacting and collaborating with several PRRs, we further examined the basal defense in *bak1 bkk1*. Three-week-old soil-grown plants of Col-0, *sid2-3, eds5-2, bak1-3, bkk1-1, bak1-3 bkk1-1, bak1-3 bkk1-1 sid2-3*, and *bak1-3 bkk1-1 eds5-2* were sprayed with *P. syringae* pv *tomato* DC3000 (*Pst* DC3000)-LUX for 30 h before the images of luciferase florescence were taken. Single mutant *sid2-3* or *eds5-2* showed enhanced susceptibility to the bacterial pathogen, indicating the essential role of SA in mediating basal defense (Figure 6A). Similarly, single mutant *bak1-3* or *bkk1-1* also exhibited markedly reduced resistance to *Pst* DC3000, consistent with the notion that *BAK1* functions as a positive regulator in PTI (Figure 6A). Surprisingly, the double mutant *bak1-3 bkk1-1* showed enhanced resistance to *Pst* DC3000, different from either *bak1-3* or *bkk1-1* (Figure 6A). Compared to *bak1-3 bkk1-1, bak1-3 bkk1-1 sid2-3* or *bak1-3 bkk1-1 eds5-2* showed reduced bacterial resistance, suggesting SA was involved in the defense activation in *bak1-3 bkk1-1*. To confirm these results, we analyzed the activation status of MPK3/6 in various plants lines. MPK3/6 were identified as mediators in basal defense and receptor triggered immunity including PTI and ETI (Rasmussen et al., 2012; Tsuda et al., 2013). Activated MPK3/6 via phosphorylation indicate enhanced immune response. By using a P-p44/42 antibody, phosphorylated MPK3/6 were detected in various plant lines. Consistent to *Pst* DC3000 treatment results, *bak1-3 bkk1-1* showed over-activated MPK3/6, which were less phosphorylated in *sid2-3, eds5-2, bak1-3, bak1-4*, or *bkk1-1*, compared to Col-0 (Figure 6B). In *bak1-3 bkk1-1 sid2-3* and *bak1-3 bkk1-1 eds5-2*, phosphorylated MPK3/6 were reduced compared to *bak1-3 bkk1-1*, suggesting the autoimmunity in *bak1-3 bkk1-1* is partially SA-dependent (Figure 6B). It was intriguing that *bak1-3* and *bkk1-1* single mutants were more susceptible but *bak1-3 bkk1-1* double mutant were more resistant to bacterial pathogen. We next analyzed PTI signaling in *bak1 bkk1* to examine whether PTI was responsible for the enhanced immunity in *bak1-4 bkk1-1*. Nine-day-old Col-0, *bak1-4 bkk1-1, bak1-4*, and *bkk1-1* were incubated with flg22, a 22-amino-acid peptide conserved in bacterial flagellin that is efficient to trigger FLS2-mediated immune signaling, for 5, 15, and 30 min before examining phosphorylated MPK3/6. Both of the single mutants *bak1-4* and *bkk1-1* showed reduced response to flg22 (Figure 6C). The response of double mutant *bak1-4 bkk1-1* to flg22 was dramatically reduced, demonstrating PTI in *bak1-4 bkk1-1* was nearly blocked (Figure 6C). Our results supported the previous findings that *BAK1* is essential for PRR-mediated PTI and, more importantly, suggested that *bak1 bkk1* double mutants undergo an enhanced immune signaling other than PTI.

### EDS1 and PAD4, components mediating ETI, were involved in the cell death in *bak1 bkk1*

**Figure 7:**
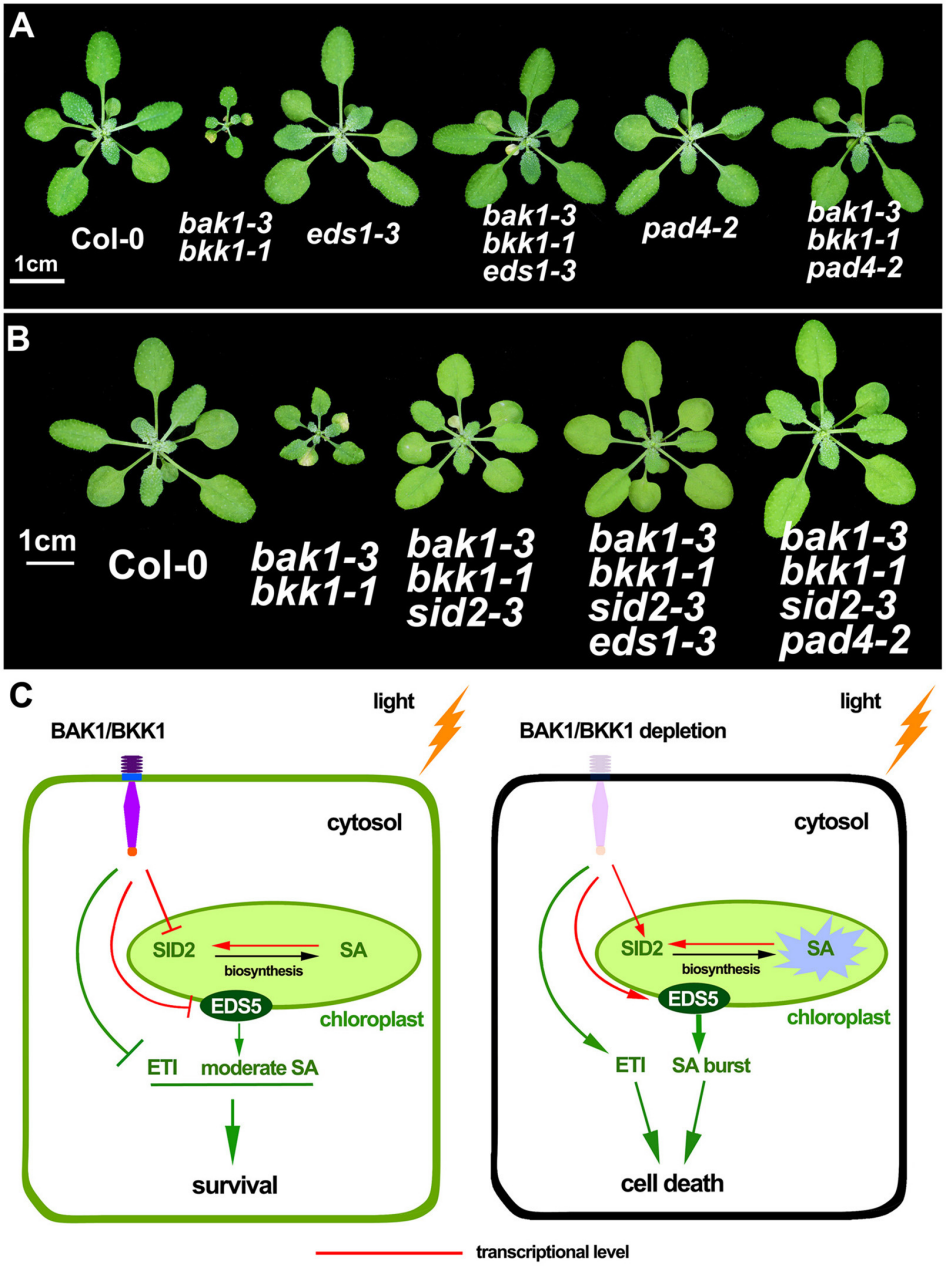
**The cell death in ***bak1-3 bkk1-1*** is significantly suppressed by the mutation in ***EDS1*** or ***PAD4*****. The representative phenotypes of 3-week-old Col-0, *bak1-3 bkk1-1, eds1-3, bak1-3 bkk1-1 eds1-3, pad4-2* and *bak1-3 bkk1-1 pad4-2* grown in soil are presented **(A)**. The representative phenotypes of 26-day-old Col-0, *bak1-3 bkk1-1, bak1-3 bkk1-1 sid2-3, bak1-3 bkk1-1 sid2-3 eds1-3*, and *bak1-3 bkk1-1 sid2-3 pad4-2* grown in soil are presented **(B)**. A hypothetical model for the cell death induction in *bak1 bkk1*. Under light condition, *BAK1* and *BKK1* repress *SID2* and *EDS5* at transcriptional levels, resulting in low concentrations of cellular SA. The absence of both *BAK1* and *BKK1* leads to highly expressed *SID2* and *EDS5*, causing overly produced endogenous SA in the chloroplast followed by SA transport to cytosol through EDS5. Positive feedback regulation of *SID2* by SA causes SA burst that ultimately triggers cell death. BAK1 and BKK1 also suppress ETI signaling mediated by EDS1-PAD4. Depletion of both BAK1 and BKK1 leads to release of the inhibition on ETI. Activated ETI induces cell death (HR) and confers plants enhanced resistance **(C)**.

EDS1-PAD4 complex acts as an essential component in mediating all tested TNL type R protein pathway in ETI (Wiermer et al., 2005). To understand whether ETI is involved in the cell death induction in *bak1 bkk1, bak1-3 bkk1-1* was crossed to *eds1-3* and *pad4-2*, respectively *eds1-3* was able to partially rescue the cell death of *bak1-3 bkk1-1*; while *pad4-2* dramatically suppressed the lesion symptom of *bak1-3 bkk1-1* (Figure 7A). Our genetic results thus implicated the autoimmunity in *bak1 bkk1* is likely ETI-related.

Given that EDS1 and PAD4 also function as upstream of SA pathway via regulating *ICS1*-generated SA biogenesis, we next tested whether the suppression of *bak1-3 bkk1-1* by *eds1* or *pad4* was SA-dependent. No clear cell death symptom was detected in 3-week-old *bak1-3 bkk1-1 sid2-3* (Figure 5). By contrast, 26-day-old *bak1-3 bkk1-1 sid2-3* started to exhibit detectable lesion symptom, suggesting the cell death pathway was partially SA-dependent. The cell death of *bak1-3 bkk1-1 sid2-3* was further suppressed by *eds1* or *pad4*, suggesting EDS1 and PAD4 contributed to the BAK1 and BKK1-mediated cell death pathway other than, or in addition to, regulating SA accumulation (Figure 7B). Therefore, ETI mediated by EDS1 and PAD4 was proposed to be involved in BAK1-mediated cell death pathway. We also introduced a mutation of *NDR1*, a key mediator in CNL signaling, into *bak1-3 bkk1-1*. The triple mutant *bak1-3 bkk1-1 ndr1-1* exhibited identical cell death phenotype to *bak1-3 bkk1-1*, suggesting TNL type but not CNL type R proteins are likely involved in BAK1 and BKK1-mediated cell death pathway (Figure S6).

## Discussion

BAK1 was originally identified as a co-receptor of BRI1, positively regulating BR signal transduction. Genetic analyses revealed that *BAK1* and its closest homolog *BKK1* can also modulate a cell death pathway that is independent of the BR signaling pathway. This unexpected finding suggested the versatile roles of BAK1 in regulating plant growth and development, as well as adaptations to environmental stresses. Removal of both *BAK1* and *BKK1* in a single plant leads to cell lesion and ultimately a lethality phenotype under a normal growth condition, indicating the irreplaceable roles *BAK1* and *BKK1* play in the physiological events essential for plants to fulfill their life cycles. However, the detailed mechanisms of *BAK1* and *BKK1* in mediating cell death are still beyond what we understand. After the finding of *BAK1-*mediated cell death, BAK1 was reported to be engaged in plant innate immunity through associating with PRRs such as FLS2, EFR and PEPR1 (Chinchilla et al., 2007; Heese et al., 2007; Schulze et al., 2010). Nevertheless, loss-of-function mutants of the BAK1-interacting PRRs only exhibit impaired innate immunity but no cell death symptom, suggesting the cell death mediated by BAK1 and BKK1 is unlikely directly related to PTI mediated by PRRs. In addition, a *BAK1* point mutation allele, *bak1-5*, was identified to display disrupted FLS2-mediated PTI, but *bak1-5 bkk1-1* showed no cell death (Schwessinger et al., 2011), suggesting that BAK1-mediated PTI pathway and BAK1-mediated cell death pathway belong to distinct signaling events.

Our previous research suggested dark growth condition largely suppressed the cell death symptom in *bak1-4 bkk1-1* and light treatment quickly induced lesion phenotype in dark-grown *bak1-4 bkk1-1*. How light serves as an initiator of cell death in *bak1 bkk1* mutants is still unclear. Furthermore, the downstream signaling components involved in cell death induction in *bak1 bkk1* are nearly unknown. Considering that PTI is not likely involved in the cell death of *bak1 bkk1*, ETI, the stronger immune response featured by quick cell death formation, is worth being investigated whether is related to *BAK1* and *BKK1*-mediated cell death pathway.

In this study, we analyzed the formation of cell death in *bak1 bkk1* upon light treatment. Our results indicated that cell death was undetectable in *bak1-4 bkk1-1* when grown in dark up to 12 days, while evident lesion symptom was induced in *bak1-4 bkk1-1* 2 days after being transferred from dark to light condition, accompanied with highly accumulated SA and ROS. Fascinatingly, dark-grown *bak1-4 bkk1-1* was capable of exhibiting obvious cell death when exogenous SA was applied, illustrating that light does not directly regulate cell death and SA might function downstream of light and play a central role in promoting the cell death in *bak1 bkk1*. qRT-PCR results revealed that two genes *SID2* and *EDS5*, critical in modulating SA biosynthesis, were significantly up-regulated in *bak1-4 bkk1-1*; and more interestingly, the protein products of both genes are located in chloroplasts. The mutation in either *SID2* or *EDS5* in *bak1-3 bkk1-1* remarkably rescued the cell death phenotype of the double mutant, suggesting chloroplast-localized SID2 and EDS5 are crucial for cell death formation in *bak1-3 bkk1-1*, likely via affecting SA accumulation. Furthermore, in a pathogen treatment assay, both *bak1-3* and *bkk1-1* single mutants were more susceptible to *Pst* DC3000 compared to Col-0, consistent to the notion that BAK1 functions as major contributor to the basal resistance to several pathogens including *Pst* DC3000 (Roux et al., 2011). Surprisingly, *bak1-3 bkk1-1* double mutant exhibited enhanced resistance to *Pst* DC3000, indicating the loss of either *BAK1* or *BKK1* causes impaired immunity but removing both *BAK1* and *BKK1* leads to triggering additional route of plant immunity instead. Genetic analyses supported this notion by showing that mutation in either *EDS1* or *PAD4*, key regulator mediating ETI, resulted in suppression of cell death in *bak1 bkk1*.

Based on the aforementioned results, we propose a hypothetic model. SA is not efficiently synthesized in *bak1 bkk1* in dark condition. Light-induced overexpression of *SID2* and *EDS5* in *bak1 bkk1* causes highly accumulated SA. Positive feedback regulation on SA biosynthetic genes by high concentrations of SA further enhances the SA biosynthesis and leads to SA burst that ultimately induce cell death in *bak1 bkk1*. In addition, BAK1 and BKK1 regulate a signaling pathway to inhibit ETI, which is mediated by EDS1-PAD4 complex, under normal growth conditions. When BAK1 and BKK1 are both depleted, ETI is activated to initiate cell death in plants, a faster and stronger defense response (Figure 7C).

Light has been reported to cause cell death when it is excessive (Szechyńska-Hebda and Karpiński, 2013); and UV light treatment often leads to lesions in plant tissues (Nawkar et al., 2013). Nevertheless, the light-dependent cell death found in *bak1 bkk1* seems to be distinct from the known light-induced cell death. The known light-induced lesion is caused by rapid ROS accumulation, a key cell death signal. In *bak1 bkk1* double mutants, the cell death symptom appears prior to the accumulation of ROS, indicating ROS is not the cause of the cell death. Instead, ROS accumulation is likely an effect of cell death in *bak1 bkk1*. Nevertheless, since ROS also serves as a feedback signal for SA biosynthesis (Torres et al., 2006), the ROS accumulation in *bak1 bkk1* may further deteriorate the cell death symptom. In addition, none of the multiple *bak1-3 bkk1-1 pif* mutant plants displayed altered lesion phenotype compared with *bak1-3 bkk1-1* (Figures S3, S4), suggesting light-induced cell death in *bak1 bkk1* is independent of *PIF*-mediated blue and red light signaling pathways (Castillon et al., 2007; Pedmale et al., 2016). Moreover, the effect of light on cell death initiation can be reproduced by SA treatment in *bak1 bkk1* in dark (Figure 3), suggesting light acts as an indirect factor to trigger cell death in *bak1 bkk1* and light signaling is not essential for this process.

Compared to those in Col-0, the transcripts of *SID2* increased about 30–40-fold in *bak1-4 bkk1-1*, demonstrating *SID2* expression is strongly inhibited by *BAK1* and *BKK1*. Even though the expression of *SID2* was indeed enhanced by light, the suppression of *SID2* by *BAK1* and *BKK1* seemed to be light-independent, indicated by similar multiple increases of *SID2* in dark-grown Col-0 and *bak1-4 bkk1-1* upon light treatment (Figure 4A). The expression of *EDS5* is suppressed by *BAK1* and *BKK1*. Compared to those in Col-0, *EDS5* transcripts increased about 2-fold in dark condition, but elevated around 12-fold 1 day after relocating to light condition in *bak1-4 bkk1-1*. This result suggested the expression of *EDS5* is synergistically controlled by light and *BAK1*and *BKK1* (Figure 4).

In PAL pathway of SA synthesis, four *PAL* genes were identified to encode enzymes facilitating production of the SA precursor in cytosol. Our results suggested the expression levels of *PAL* genes were also inhibited by *BAK1* and *BKK1*. It may explicate that high total SA content was detected under dark condition in *bak1-4 bkk1-1*. The *PAL*-dependent SA biosynthesis pathway may contribute to the cell death initiation in *bak1-4 bkk1-1* when grown in dark for an elongated period of time (Figures S1, S2). It is also possible that additional BAK1 and BKK1-mediated pathway that is SA-independent may contributes to the cell death signaling. Despite high concentration of total SA in *bak1-4 bkk1-1* in dark, the free SA content was almost equal to that in Col-0. Thus, *BAK1* and *BKK1* might control an unknown SA regulation pathway conjugating SA to other components, serving as a mechanism of preventing toxicity caused by highly accumulated free SA (Dempsey et al., 2011).

A recent study indicated that *bak1* mutant exhibited enhanced resistance to *Pst* DC3000, through reprogramming PEPR-mediated defense signaling. Upon *Pst* DC3000 treatment, *PROPEP* genes were up-regulated at 24 hpi (hours post-inoculation) and *bak1* was more resistant at 3 dpi (days post-inoculation) (Yamada et al., 2016). It suggested the resistance to *Pst* DC3000 found in *bak1* is caused by activation of an additional immune response, the PEPR-mediated signaling. To wipe out the potential interference by secondary response in *bak1*, we examined the early responses of various plant lines to *Pst* DC3000 at 30 hpi. Instead, the single mutant *bak1-3* or *bkk1-1* was more susceptible to *Pst* DC3000, which is in line with previous studies showing that BAK1 serves as a co-receptor for multiple PRRs and the mutation of *BAK1* causes impaired PTI and reduced resistance (Chinchilla et al., 2007; Heese et al., 2007; Schulze et al., 2010; Roux et al., 2011; Schwessinger et al., 2011). It is interesting that *bak1-3 bkk1-1* double mutant was more resistant to *Pst* DC3000 (Figure 6A). In an flg22 treatment assay, we confirmed that the sensitivity of flagellin PAMP was almost abolished in *bak1-4 bkk1-1* (Figure 6C). Thus, *bak1 bkk1* double mutant maintains a dampened PTI but exhibits enhanced pathogen resistance. Therefore, additional immune signaling must be activated in *bak1-3 bkk1-1*. Our results showed that *eds1* or *pad4* can significantly suppress the cell death phenotype of *bak1-3 bkk1-1*, supporting the notion that ETI is inhibited by BAK1 and BKK1-mediated signaling (Figure 7A). It explained why *bak1-3 bkk1-1* was more resistant to pathogen. Although EDS1-PAD4 complex was known to regulate SA via affecting *ICS1*-generated SA biosynthesis, the suppression of *bak1-3 bkk1-1* by *eds1* or *pad4* appears to be SA-independent. *bak1-3 bkk1-1 sid2-3* showed lesion phenotype when grown for an extended period of time. *eds1* or *pad4* suppressed the cell death of *bak1-3 bkk1-1 sid2-3*, suggesting EDS1-PAD4 regulate BAK1-mediated cell death signaling via a SA-independent manner (Figure 7B). In a recent report, it was shown that the major function of EDS1 and PAD4 in mediating ETI is SA-independent (Rietz et al., 2011). This study was in line with our results showing that *sid2* only partially suppressed the cell death of *bak1-3 bkk1-1* and that *eds1* or *pad4* further enhanced the suppression of cell death in *bak1-3 bkk1-1 sid2-3*.

Pathogen-derived effectors target key components mediating PTI in plant cell. FLS2, EFR, or CERK1 (CHITIN ELICITOR RECEPTOR KINASE 1) was shown to be the substrate of several effectors such as Pto, a kinase inhibitor, PtoB, a ubiquitin ligase, and HopAO1, a tyrosine phosphatase (Göhre et al., 2008; Xiang et al., 2008; Gimenez-Ibanez et al., 2009; Macho et al., 2014). The association between PRRs and effectors leads to dysfunction or degradation of PRRs, a strategy of pathogens to suppress plant immunity and enhance pathogenesis. BAK1, the co-receptor of several key PRRs, is also the target of several effectors, including PtoB, HopF2, an ADP-ribosyltransferase, and HopB1, a protease (Shan et al., 2008; Zhou et al., 2014; Li et al., 2016). It seems to be an efficient way for invading pathogens to attack a co-receptor in order to interrupt multiple PRR-mediated immune signalings. In our model, BAK1 and BKK1 may serve as guard proteins to surveil pathogen invasions. Depletion of BAK1 and BKK1 caused by effector attacks activates ETI, the stronger immunity featured by induction of cell death (HR), to enhance plant resistance to pathogens (Figure 7C).

In this study, we investigated the mechanism of how light functions as a primary factor in initiating cell death in *bak1 bkk1*. Our results supported light-induced overly expressed SA biosynthetic genes play a central role in this process. We also found key ETI components contribute to the cell death formation in *bak1 bkk1*. Future studies will focus on whether R proteins, the initiators of ETI signaling, function as downstream substrates of BAK1 and BKK1 and are engaged in the cell death induction in *bak1 bkk1*.

## Author contributions

KH, JL, and YG designed the experiments. YG, YW, JD, YZ, DS, JZ, and SZ performed the experiments. KH and YG analyzed the data and wrote the manuscript.

### Conflict of interest statement

The authors declare that the research was conducted in the absence of any commercial or financial relationships that could be construed as a potential conflict of interest.
