# Supplementary material for: Both Light-Induced SA Accumulation and ETI Mediators Contribute to the Cell Death Regulated by BAK1 and BKK1
Source: Front Plant Sci. 2017 Apr 25;8:622. doi: 10.3389/fpls.2017.00622 (PMC5403931; doi:10.3389/fpls.2017.00622)
Supplement: Supplementary file 1 [file Table1.DOCX]

**Table S1.** Primers used for gene expression analysis.

| Name | Sequence | |
| --- | --- | --- |
| PR1-QRTF  PR1-QRTR  FMO1-QRTF  FMO1-QRTR  SID2-QRTF  SID2-QRTR  EDS5-QRTF  EDS5-QRTR  PAL1-QRTF  PAL1-QRTR  PAL2-QRTF  PAL2-QRTR  PAL3-QRTF  PAL3-QRTR  PAL4-QRTF  PAL4-QRTR  BAK1-RTF  BAK1-RTR  BKK1-RTF  BKK1-RTR  SID2-RTF  SID2-RTR  EDS5-RTF  EDS5-RTR  EDS1-RTF  EDS1-RTR  PAD4-RTF  PAD4-RTR | | CATACACTCTGGTGGGCCTTA  CGCTAACCCACATGTTCACG  CTCTTCTGCGTGCCGTAGTTT  TCCCTTTATCCGCTTCCTCAA  ACCAGCAAATCGGAGCAA  AGAAGATCGGGACGACCAA  GTGGCCGTTTATCCTTGTTG  AATGATCGTTGCTGCAGCTA  GCTTAAGGCAGTGCTACCGAA  GAACTCTTCTCCAGGCGACGT  TGTGAAGGTAAACTTATTGATCCGT  TCAATTCAAACATTAACACAGCAAA  CGACACGGTTTTTCGGAAAAT  ACCGGTACAGCGGATACGATC  TCCACAAAATCGGAGCTTTTG  TCAAACTCCTCTCCTGGCGAC  ATGGAACGAAGATTAATGATCCCT  TAATATGGAGCAGCAGAAGC  TTTGCTGTTCCAGCCATTGCGT  GCCAGCATGAGGATAGGCTTGA  GGGCTCAAACACTAAAACACA  TCTTCCTTCGTAAGTCTCCCT  ATTCGGTCCTTGGGCTGTTAC  GCAGCCGAGTAAACCATAGCC  GAAGAAGCAGGAGCAGTCGTA  GGCTCAACTAATCTGCGGTAT  ATGGACGATTGTCGATTCGA  ATCGTAGTAACCTAGCTGCTCTTCT |
