# Supplementary material for: Both Light-Induced SA Accumulation and ETI Mediators Contribute to the Cell Death Regulated by BAK1 and BKK1
Source: Front Plant Sci. 2017 Apr 25;8:622. doi: 10.3389/fpls.2017.00622 (PMC5403931; doi:10.3389/fpls.2017.00622)
Supplement: Supplementary file 2 [file DataSheet1.DOCX]

**Figure S1 |** ***BAK1* and *BKK1* down-regulate *PAL* genes.** Quantitative RT-PCR assays indicate increased expression levels of four *PAL* genes in *bak1-4 bkk1-1* compared to Col-0. *PAL1***(A)**, *PAL2***(B)**, *PAL3***(C)** and *PAL4***(D)** are analyzed in the seedlings grown in dark (D) for 4 days then relocated to long-day (L) condition for indicated days (4D+0L, 4D+1L and 4D+2L).

**Figure S2 |** **Cell death is triggered in *bak1-4 bkk1-1* grown in dark for elongated periods.** Seedlings grown in dark (D) for the indicated days (9D+0L, 10D+0L, 11D+0L, 12D+0L, 15D+0L) are presented. The trypan blue staining assays indicate the cell death symptom begins to be detectable in *bak1-4 bkk1-1* grown in dark after 12 days **(A)**. The DAB staining assays indicate ROS accumulation becomes obvious in *bak1-4 bkk1-1* grown in dark after 15 days **(B)**. The scale bars represent 100µm.

**Figure S3 |** ***BAK1* and *BKK1*-mediated cell death control is independent of *PIF1* and *PIF3*.** The representative phenotypes of three-week-old Col-0, *bak1-3* *bkk1-1*, *pif1-1, pif3-1,* *pif1-1 pif3-1*, *bak1-3 bkk1-1 pif1-1, bak1-3 bkk1-1 pif3-1* and *bak1-3 bkk1-1 pif1-1 pif3-1* grown in soil are presented **(A)**. The mutations of *PIF1* and *PIF3* do not cause phenotypic difference compared to *bak1-3 bkk1-1* with regard to cell death **(A)**. Trypan blue staining **(B-I)** and DAB staining **(J-Q)** assays indicate cell death and ROS accumulation are identical in *bak1-3 bkk1-1, bak1-3 bkk1-1 pif1-1, bak1-3 bkk1-1 pif3-1* and *bak1-3 bkk1-1 pif1-1 pif3-1*. Quantitative RT-PCR assays indicate the expression levels of *PR1* and *FMO1* are similar in *bak1-3 bkk1-1, bak1-3 bkk1-1 pif1-1, bak1-3 bkk1-1 pif3-1* and *bak1-3 bkk1-1 pif1-1 pif3-1* **(R-S)**. Scale bars represent 1cm **(A)** and 100µm **(B-Q)**, respectively. 11-day-old seedlings are analyzed in **(B-S)**.

**Figure S4 | *BAK1* and *BKK1*-mediated cell death control is independent of *PIF4* and *PIF5*.** The representative phenotypes of three-week-old Col-0, *bak1-3* *bkk1-1*, *pif4-3, pif5-3,* *pif4-3 pif5-3*, *bak1-3 bkk1-1 pif4-3, bak1-3 bkk1-1 pif5-3* and *bak1-3 bkk1-1 pif4-3 pif5-3* grown in soil are presented **(A)**. The mutations of *PIF4* and *PIF5* do not cause phenotypic difference compared to *bak1-3 bkk1-1* with regard to cell death **(A)**. Trypan blue staining **(B-I)** and DAB staining **(J-Q)** assays indicate cell death and ROS accumulation are identical in *bak1-3* *bkk1-1*, *bak1-3 bkk1-1 pif4-3, bak1-3 bkk1-1 pif5-3* and *bak1-3 bkk1-1 pif4-3 pif5-3*. Quantitative RT-PCR assays indicate the expression levels of *PR1* and *FMO1* are similar in *bak1-3 bkk1-1, bak1-3 bkk1-1 pif4-3, bak1-3 bkk1-1 pif5-3* and *bak1-3 bkk1-1 pif4-3 pif5-3* **(R-S)**. Scale bars represent 1cm **(A)** and 100µm **(B-Q)**, respectively. 11-day-old seedlings are analyzed in **(B-S)**.

**Figure S5 |** **Identifications of mutant alleles.** Semi-quantitative RT-PCR assays indicate *bkk1-1*, *sid2-3*, *eds5-2*, *eds1-3*, *pad4-2* are knockout mutants. *bak1-3* is a weak mutant allele expressing lower levels of *BAK1* in corresponding genetic lines **(A-D)**.

**Figure S6 |** ***BAK1* and *BKK1*-mediated cell death control is independent of *NDR1.*** The representative phenotypes of three-week-old Col-0, *bak1-3* *bkk1-1*, *ndr1-1*, *bak1-3 bkk1-1 ndr1-1* grown in soil are presented.

**Table S1. Primers used for gene expression analysis.**
