# Supplementary figures and images for: Both Light-Induced SA Accumulation and ETI Mediators Contribute to the Cell Death Regulated by BAK1 and BKK1
Source: Front Plant Sci. 2017 Apr 25;8:622. doi: 10.3389/fpls.2017.00622 (PMC5403931; doi:10.3389/fpls.2017.00622)

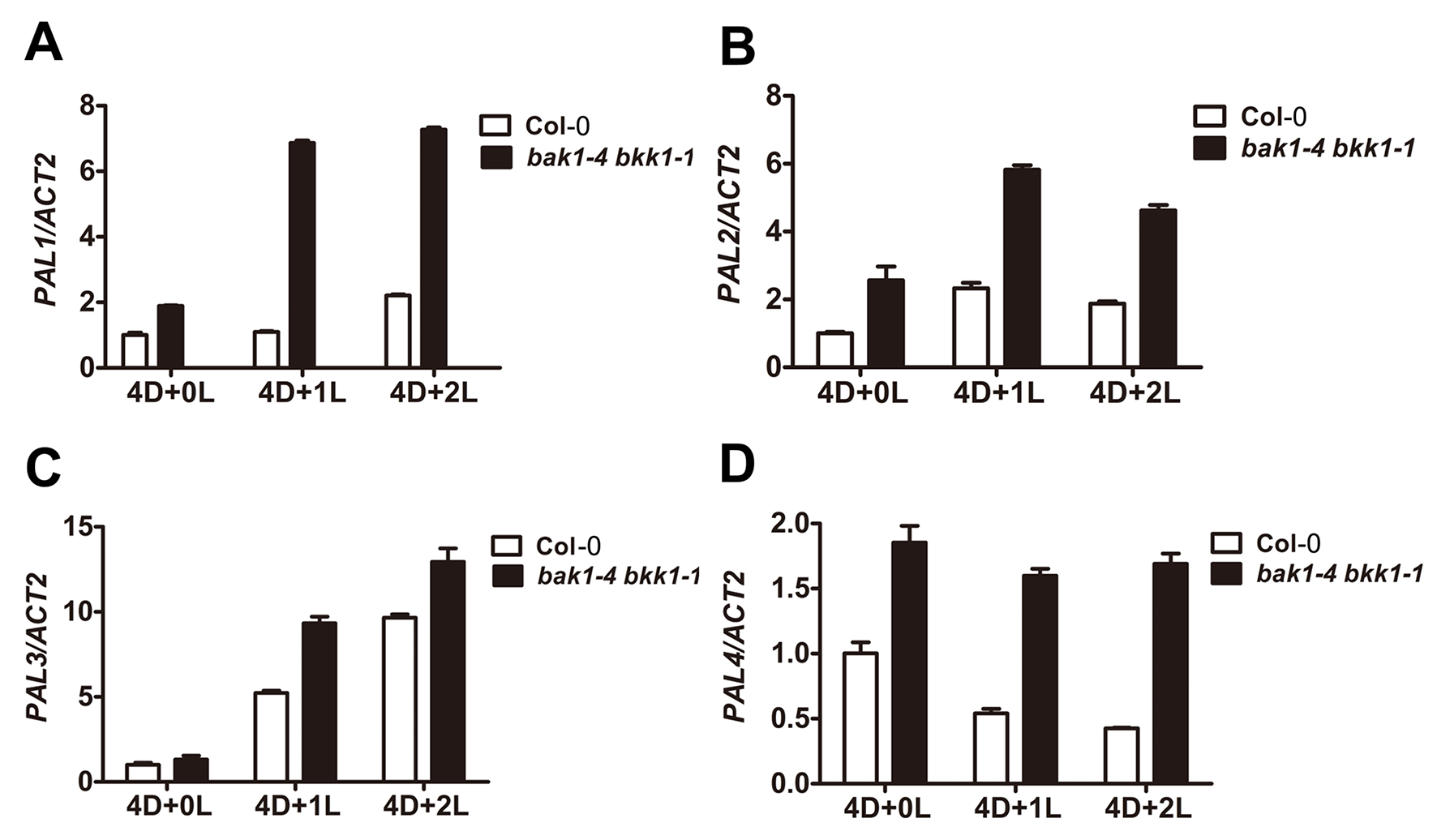

Supplement: Supplementary file 3 [file Image1.TIF]

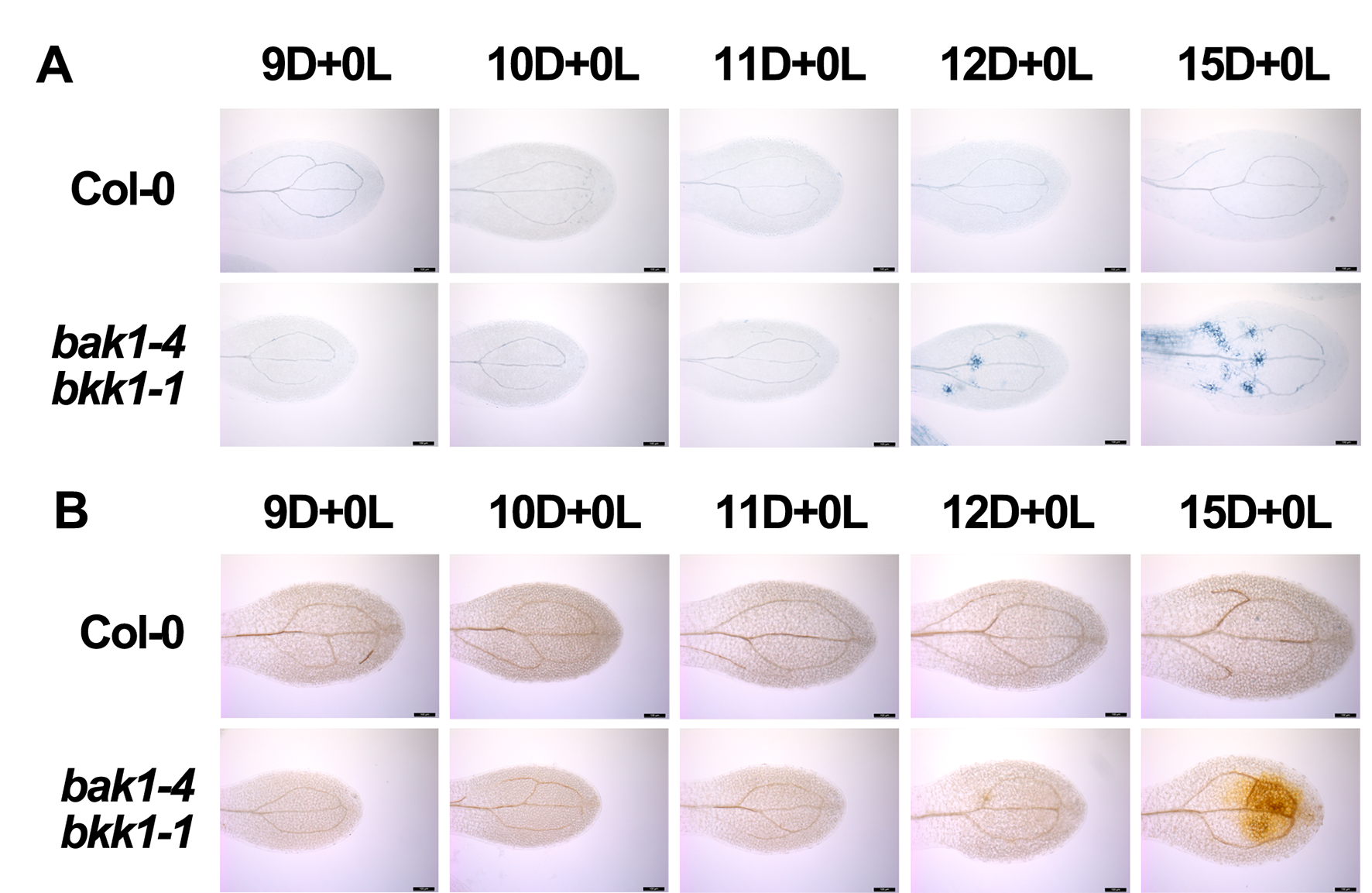

Supplement: Supplementary file 4 [file Image2.TIF]

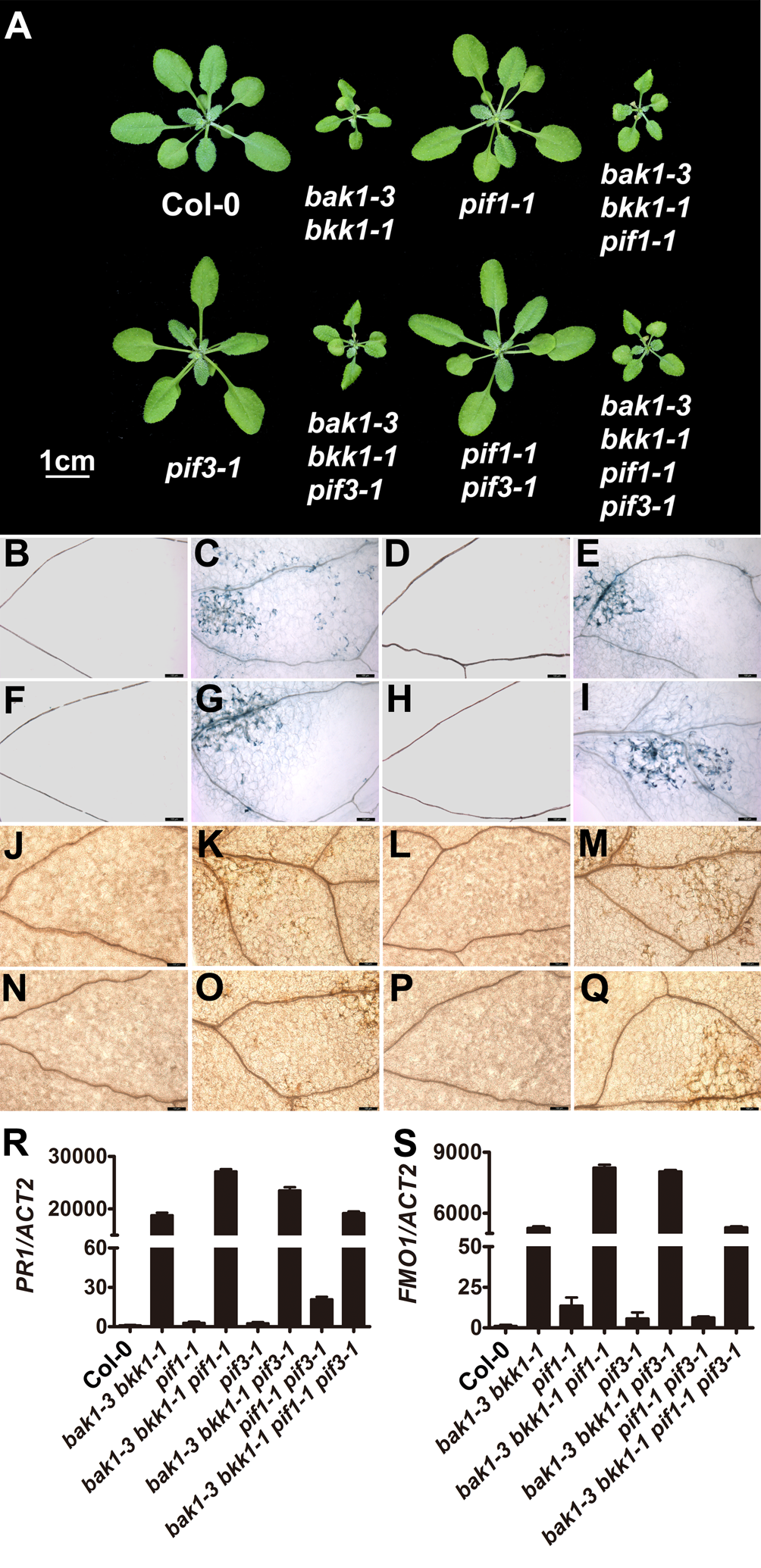

Supplement: Supplementary file 5 [file Image3.TIF]

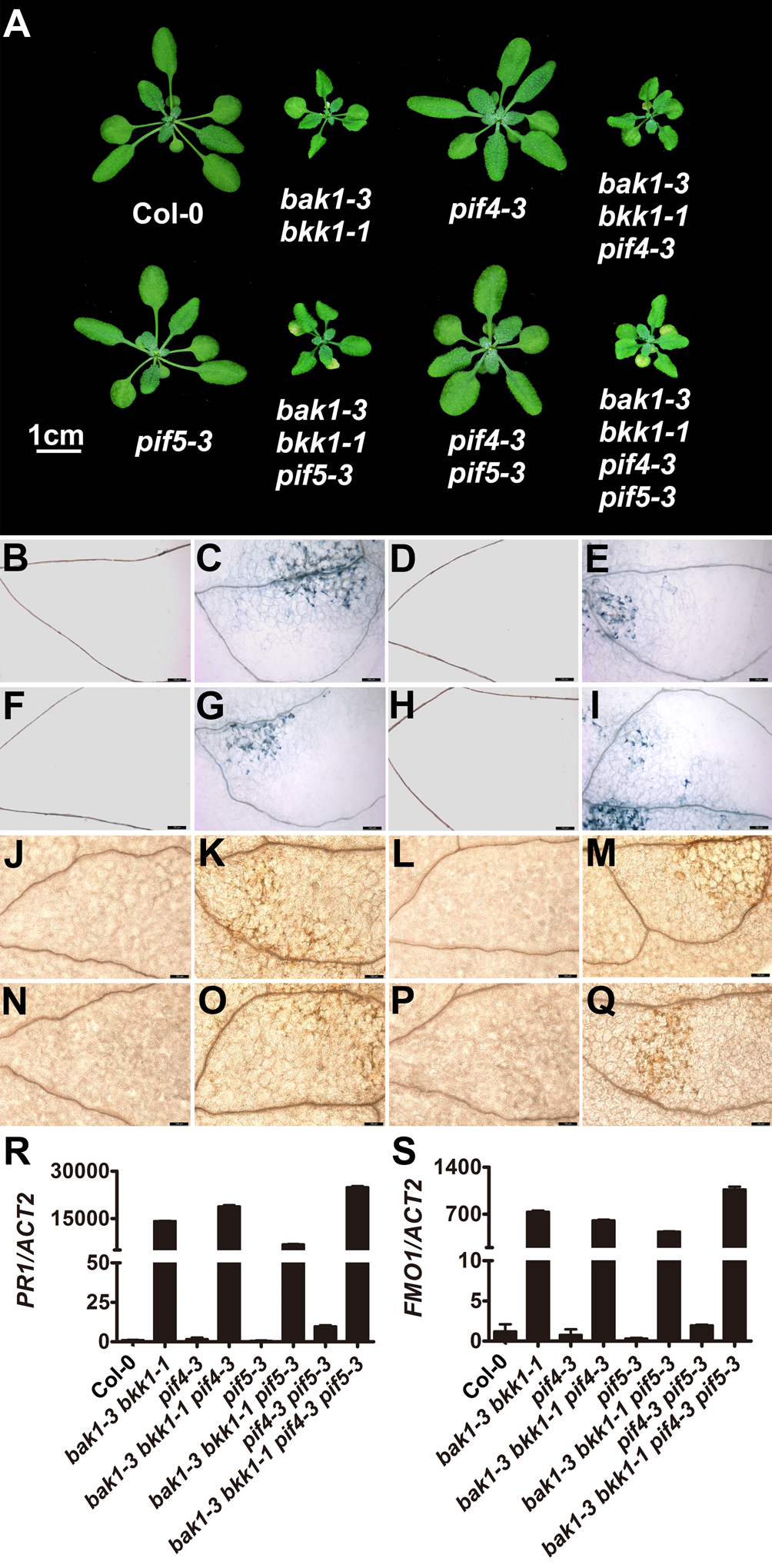

Supplement: Supplementary file 6 [file Image4.TIF]

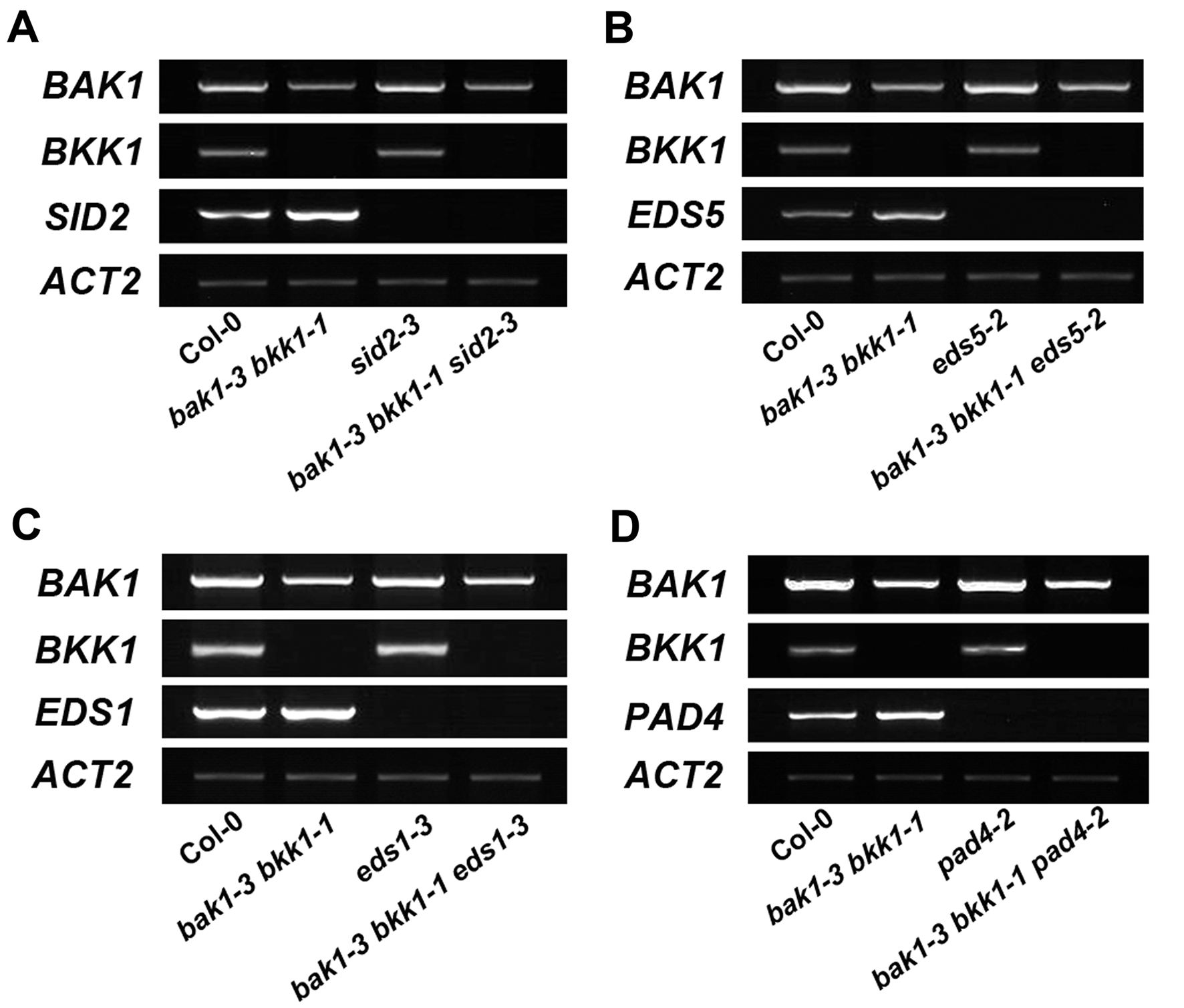

Supplement: Supplementary file 7 [file Image5.TIF]

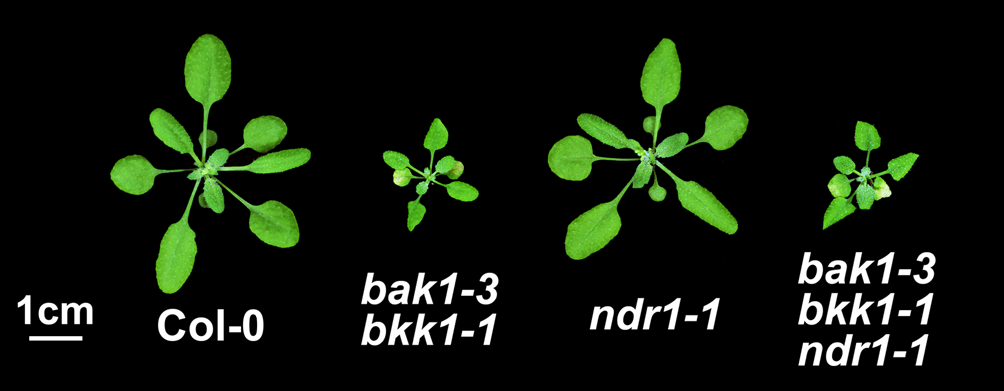

Supplement: Supplementary file 8 [file Image6.TIF]
